# Foreign-body aspiration into the lower airways in adults; multicenter study

**DOI:** 10.1371/journal.pone.0269493

**Published:** 2022-07-06

**Authors:** Gimun Jang, Jae Woon Song, Hyun Jung Kim, Eun Jin Kim, Jong Geol Jang, Seung-Ick Cha

**Affiliations:** 1 Department of Medicine, School of Medicine, Keimyung University, Daegu, South Korea; 2 Division of Pulmonary and Critical Care Medicine, Department of Internal Medicine, Keimyung University Dongsan Hospital, Daegu, South Korea; 3 Department of Internal Medicine, School of Medicine, Daegu Catholic University, Daegu, South Korea; 4 Division of Pulmonary and Critical Care Medicine, Department of Internal Medicine, Regional Respiratory Center, Yeungnam University Hospital, Daegu, South Korea; 5 Division of Pulmonary and Critical Care Medicine, Department of Internal Medicine, School of Medicine, Kyungpook National University, Daegu, South Korea; University Hospital of Modena (Italy), Respiratory Diseases Unit, ITALY

## Abstract

**Background:**

Foreign-body aspiration is common in children aged 6 months to 3 years. However, with the aging population and increasing prevalence of disabilities such as hemiparesis and neuromuscular diseases in adults, an increased incidence of aspiration is expected.

**Methods:**

This was a multicenter retrospective, observational study in four major referral hospitals in Daegu, South Korea, between 2000 and 2019. We included patients aged over 18 years who were evaluated for tracheobronchial foreign-body aspiration by flexible bronchoscopy. Comorbidities, type and location of foreign body, and radiologic findings were recorded.

**Results:**

Of 138 patients who underwent flexible bronchoscopy for tracheobronchial foreign body aspiration, 91 (65.9%) were men; the mean age was 66.3 (range: 29–87) years. A history of definite choking was present in 60 (43.5%) patients. The most common site of the foreign body was the right bronchus intermedius (27.5%). The most common type of aspirated foreign body was teeth (37.7%), followed by chicken bone (15.2%), nuts (14.5%) and fish bone (9.4%). Iatrogenic events accounted for 37.0% of the cases of aspiration, and the foreign body was successfully removed by flexible bronchoscopy in 91.3% of cases.

**Conclusion:**

Foreign-body aspiration is not rare, even in adults who do not have predisposing factors. Iatrogenic events accounted for about 40% of all cases of foreign body aspiration. In adults, flexible bronchoscopy is relatively safe and has a high success rate for foreign-body removal.

## Introduction

Tracheobronchial foreign bodies (TFBs) may be found incidentally or present with life-threatening symptoms that require immediate intervention [1, 2]. Although aspiration into the lower airways is common among children aged 5 years or less, it is rare in adults [3–5]. Alcohol intoxication, sedative or hypnotic drug use, primary neurologic disorders, and seizure are predisposing factors for TFBs [6, 7].

Previous studies of TFBs in adults reported that the type of foreign body differed according to the medical environment and table manners [4, 7]. The most common TFBs were metallic (41%) and organic (25.6%) in India [8]; chicken bone and fish bone in China [5]; and food and garden peas in Belgium [1]. However, no data are available for Korean adults.

TFBs are not common in adults; however, aspiration is often seen in clinical practice. We hypothesized that the aging of the population and prolonged survival of patients with disabilities may lead to an increase in the incidence of TFBs, and alter the type thereof and predisposing factors. No data have been published from Korea regarding hospital visits for TFBs or TFB types in adults. Therefore, the aim of this study was to retrospectively analyse TFB types, and their predisposing factors, clinical presentation, and treatment methods in adults.

## Methods

Medical records were retrospectively collected from four major referral hospitals in Daegu, South Korea, for the period 2000–2019. We excluded patients aged less than 18 years and those with broncholith or bronchial tumors. We recorded the demographic information, choking history, mental status, daily performance status, type of foreign body, radiologic findings, and location of foreign body for 138 patients. The patients were categorized into those with acute, subacute, chronic, or uncertain TFB. Acute TFB was diagnosed on bronchoscopy within 7 days of the aspiration, subacute within 7–30 days of aspiration, and chronic 30 days or more after aspiration. In the absence of a definite aspiration event, we defined TFB as uncertain. Differences between acute and chronic groups were analysed. Relationship of age and predisposing factor were analysed using chi-square test.

All patients underwent elective or emergent bronchoscopy for reasons such as aspiration, foreign body on imaging, and chronic cough. Flexible bronchoscopy was performed under light sedation administered through the mouth or a previously inserted endotracheal tube, with spontaneous breathing or mechanical ventilation. Foreign-body removal was attempted using suction, forceps, or a wire basket inserted through the bronchoscope channel. In case of failure, additional procedures, such as rigid bronchoscopy, surgery, or transfer, were performed. After the aspirated material had been removed, patients were monitored for adverse events.

This study was approved by the Institutional Review Board of Keimyung University Dongsan Medical Center, Korea (no. 2020-09-057).

## Results

### Baseline characteristics

The study participants included 91 male patients (65.9%) and the mean age was 66.3 ± 11.6 years (range: 29–87 years). Sixty patients (43.5%) had a history of aspiration and most (73.9%) were able to perform activities of daily living independently. No predisposing factor was detected in 97 patients (70.3%), while the most common predisposing factor was cerebrovascular accident in 19 patients (13.8%), followed by traumatic loss of consciousness, Parkinson’s disease, and seizures. At the time of bronchoscopy, 111 patients (80.4%) were alert. The most common location of the aspiration was at home (25.4%), followed by dental clinics (21.0%), nursing hospitals (8.0%), hospitals (7.2%), and accident sites (3.6%). Table 1 summarizes the baseline characteristics of the participants.

**Table 1:** Demographic characteristics of the patients (N = 138).

Parameters	Values
Age	66.3 ± 11.6 (29–87)
Male	91 (65.9)
History of aspiration	60 (43.5)
Mental status	
alert	111 (80.4)
not alert	27 (19.6)
Impairment of protective airway mechanism	41 (29.7)
alcohol or sedatives	2 (1.4)
brain tumor	1 (0.7)
cerebrovascular accident	19 (13.8)
dementia	2 (1.4)
mental retardation	2 (1.4)
Parkinson disease	3 (2.2)
seizure	3 (2.2)
traumatic loss of consciousness	4 (2.9)
Etc.*	5 (3.6)
Daily activity	
without any help	102 (73.9)
cane / wheelchair	17 (12.3)
bed-ridden	19 (13.9)
Place of aspiration event	
home	35 (25.4)
dentistry	29 (21.0)
nursing hospitals	11 (8.0)
in-hospital	10 (7.2)
accident	5 (3.6)
Chest CT scan	127(92.0)

Data are presented as n (%) or mean ± SD (range).

* Etc. includes intubation, hanging and psychosis.

### Clinical presentation and radiologic findings

The most common presenting symptom of TFB was cough (60.9%), and 61 patients (44.2%) had dyspnea. Other clinical presentations were fever (23.9%), blood-tinged sputum (17.4%), and wheezing (15.9%). Supplemental oxygen was required in 43 patients (31.2%) at the time of presentation.

Table 2 summarizes the radiologic findings of the study participants. Foreign-body opacity was seen in 51 (37.0%) of the 138 cases. The chest radiograph showed pneumonia in 37 patients (26.8%), atelectasis in 23 (16.7%), obstructive emphysema in 1 (0.7%), and normal findings in 26 (18.8%).

**Table 2:** Radiographic findings in patients with tracheobronchial foreign bodies.

Radiologic findings	N (%)
Foreign body opacity	51 (37.0)
Pneumonic infiltration	37 (26.8)
Normal	26 (18.8)
Atelectasis	23 (16.7)
Obstructive emphysema	1 (0.7)

Chest CT scans were taken in 127 (92.0%) patients before performing bronchoscopy and TFBs were found in 78 (60.9%) chest CT scans. In 29 (22.8%) cases, no obvious foreign body was observed on chest CT scan, but CT findings such as suspicious endobronchial lesion, mucus and atelectasis indicated the need for bronchoscopy.

### Bronchoscopic findings

Emergency bronchoscopy was performed in 59 (42.8%) of 138 patients; ventilator support was required for 14 patients (10.1%) during bronchoscopy. Table 3 lists the reasons for bronchoscopy. The most common indication for bronchoscopy was aspiration (39.1%), followed by foreign body on imaging (29.0%), suspicious endobronchial lesion (13.0%), chronic cough (8.7%), recurrent pneumonia (5.8%), and hemoptysis (3.6%).

**Table 3:** The indication of bronchoscopy.

Reason of bronchoscopy	N (%)
Aspiration history	54 (39.1)
Foreign body on image	40 (29.0)
Suspicious endobronchial lesion on image	18 (13.0)
Unexplained chronic cough	12 (8.7)
Recurrent pneumonia	8 (5.8)
Haemoptysis	5 (3.6)
Suspicious bronchopulmonary fistula	1 (0.7)

**Fig 1:**
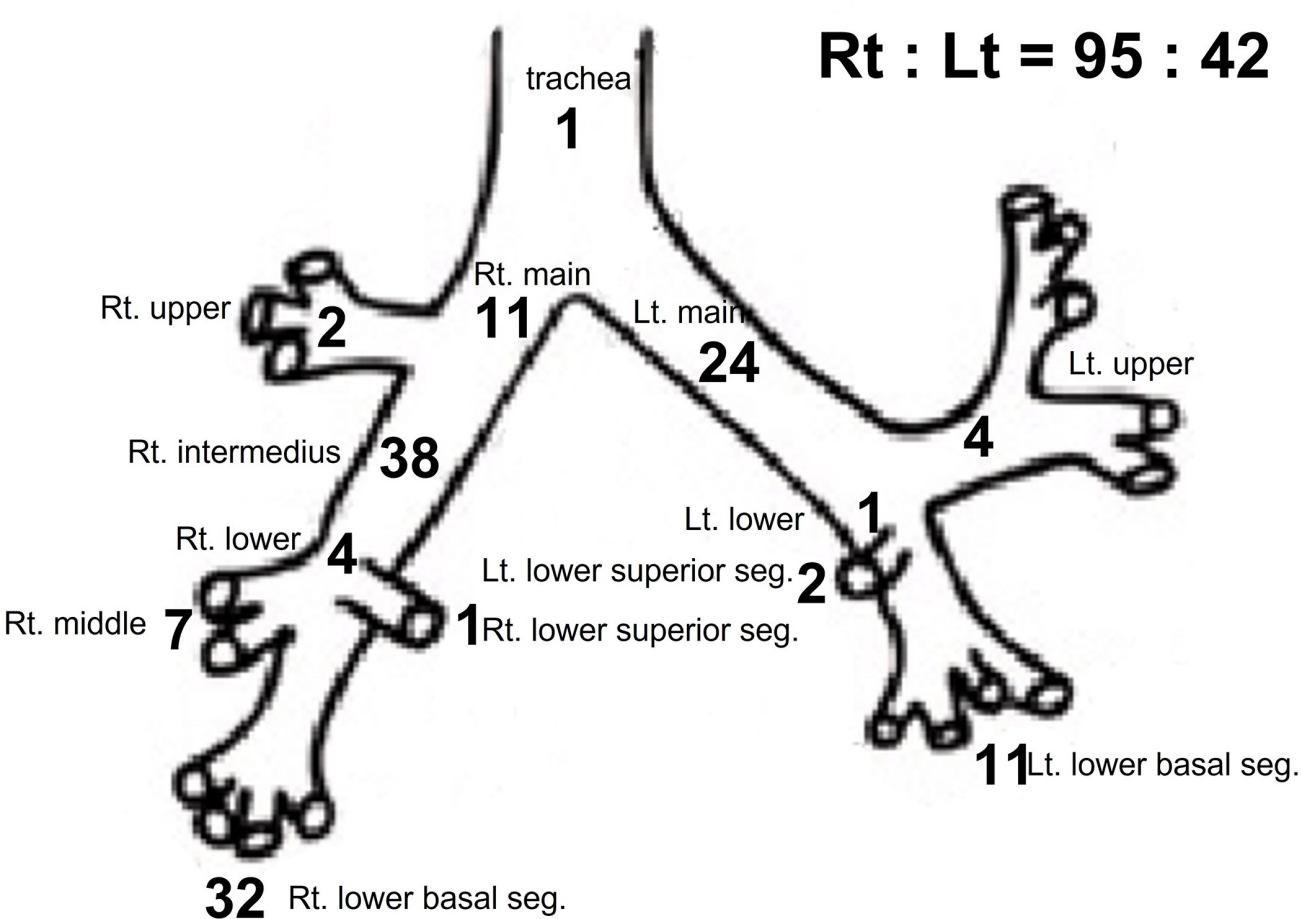
Distribution of the location of foreign body in bronchus. (Total N = 138).

The foreign bodies were located in the trachea in 1 patient (0.7%), right bronchial tree in 95 patients (68.8%), and left bronchial tree in 42 patients (30.4%) (Fig 1). In total, 38 foreign bodies (27.5%) were located in the right bronchus intermedius and 32 (23.2%) in the right lower lobe basal segment.

### Relationship of age and predisposing condition

Among 36 patients aged less than 60 years, 16 (44.4%) had predisposing factors, such as mental retardation, traumatic loss of consciousness, or stroke. However, among the 102 patients aged 60 years or older, 27 (26.5%) had risk factors for aspiration. (p = 0.073) (Table 4).

**Table 4:** Relationship of age and predisposing factors.

	Age < 60	Age ≥ 60	P-value
With predisposing factor	16 (44.4)	27 (26.5)	0.073
Without predisposiong factor	20 (55.6)	75 (73.5)	

Presented as N (%)

### Comparison of patients with acute and chronic aspiration

Acute, subacute, and chronic aspirations were detected in 60 (43.5%), 19 (13.8%), and 16 (11.6%) patients. Forty-three (31.1%) patients had no history of aspiration.

In the acute group, 42 patients (70.0%) had a history of aspiration and underwent emergency bronchoscopy, whereas 39 patients (65.0%) had a radiopaque foreign body on imaging. In the acute group, the most common foreign-body type was tooth or dental prosthesis (53.3%), followed by chicken or fish bones (33.3%) and pills (8.3%).

In the chronic group, only 1 patient underwent emergency bronchoscopy, and 15 (93.8%) underwent elective bronchoscopy. In the chronic group, only two patients had a history of aspiration and the most common indications for bronchoscopy were recurrent pneumonia and unexplained chronic cough (56.3%). Suspicious endobronchial lesions were noted on the chest radiograph of three patients.

### Foreign-body types

Table 5 summarizes the TFB types. The most common TFB material was tooth (37.7%), followed by chicken bone (15.2%), nuts (14.5%) and fish bone (9.4%). Iatrogenic causes of aspiration were found in 51 patients (37.0%), and included dental procedures, intubation, and tracheostomy tube change.

**Table 5:** The type of tracheobronchial foreign bodies.

Aspirated material	N (%)
Tooth	52 (37.7)
Chicken bone	21 (15.2)
Nuts, seeds, bean and cone	20 (14.5)
Fish bone	13 (9.4)
Food particle	12 (8.7)
Unknown	7 (5.1)
Pills	5 (3.6)
Tracheostomy tube	2 (1.4)
Etc.	6 (4.3)

*Etc. includes a cotton swab, a Go stone, a nail, a nut, a drawing pin and a piece of rubber

### Outcome of flexible bronchoscopic removal of foreign-bodies

Flexible bronchoscopic foreign-body removal was successful in 91.3% of patients. Most patients experienced no adverse events. After bronchoscopy, minor bleeding occurred in five patients (3.6%) and fever occurred in one patient (0.7%). Of the 126 cases in which foreign bodies were successfully removed by flexible bronchoscopy, bronchoscopic forceps were used in 84 cases, and wire baskets were used in 21 cases. Of the 12 cases in which flexible bronchoscopy failed to remove the foreign body, foreign bodies were removed spontaneously by coughing in two cases. One patient underwent rigid bronchoscopy, two underwent surgery, and two were transferred to other hospitals. Three remained without additional treatment, and two had lost follow-up.

## Discussion

The incidence of TFB among adults is relatively low. We collected data from four major university hospitals in our city and identified 138 cases of TFB in the past 20 years. In our study, iatrogenic causes of aspiration were seen in 37.0% of patients and the most common aspirated materials were teeth. Additionally, most TFBs (91.3%) were successfully removed by flexible bronchoscopy without serious adverse events.

We expect that the incidence of TFB may increase due to an increase in predisposing conditions, such as stroke, Parkinson’s disease, and motor neuron disease. However, people with no predisposing factors accounted for 70.3% of the cases in this study. Even healthy people are at risk of aspiration during dental treatment, and the risk is increased by local anesthesia, a supine position, and decreased cough reflex because of sedation [9]. In this study, teeth were the most common identified foreign material, which explains why people without risk factors for aspiration had to undergo bronchoscopy because of TFBs. Due to the aging of society, the number of dental treatments may increase. Moreover, the increase in the number of implant procedures, which are popular in Korea and increase the risk of aspiration, may also contribute to an increase in TFBs during dental treatment [10–12].

While the relationship of age and predisposing factor was statistically insignificant in this study, foreign-body aspiration may occur during seizure, or due to trauma or predisposing condition in relatively young people. On the other hand, even those without predisposing factors, aspiration may occur more frequently in elderly people. This is, in contrast to previous reports that many young patients accidentally aspirate small plastic or metal objects introduced into the mouth [13].

The clinical manifestations of TFB are variable and nonspecific [5]. In our study, 20 patients (14.5%) did not have cough, wheeze, bloody sputum, or dyspnea. A history of aspiration was not found in 60 patients (43.5%). The diagnosis is usually clear in cases of choking or an aspiration history. However, in the absence of such a history, and among patients who cannot verbally explain their symptoms, the correct diagnosis cannot be made. Occult TFBs in adults can remain undetected for years, leading to incorrect diagnoses [14, 15]. In this study, recurrent pneumonia and unexplained cough were the most common indications for bronchoscopy in the chronic TFB group. Foreign body aspiration occurs infrequently in adults and has nonspecific clinical manifestations; therefore, a high index of clinical suspicion is required to make the correct diagnosis. Patients with unexplained chronic respiratory symptoms (hemoptysis, cough, or recurrent pneumonia) should undergo bronchoscopy, even if the chest radiograph is normal and there is no history of choking, because TFBs may cause nonspecific chronic respiratory symptoms in adults without predisposing factors [15, 16].

Radiopaque foreign bodies were found in 37.0% of the patients in this study, a higher rate than in previous studies, which reported radiopaque foreign bodies in 10–20% of patients [17, 18]. Many types of radiopaque materials may be aspirated, including implant screws and dental and medical devices. Most iatrogenic TFBs are radiopaque; chest radiography is a non-invasive and useful tool for the diagnosis of TFB. Even if the foreign body is not radiopaque, chest radiographs may reveal signs such as atelectasis or obstructive pneumonitis, which are indications for bronchoscopy. Chest CT scan showed almost twice the foreign body detection rate compared to simple chest radiography in this study. Chest CT scan s not only useful for finding non-radio-opaque TFBs, but also for finding complications of TFBs such as aspiration pneumonia, pneumomediastinum and bronchiectasis [19].

The most common TFB type in this study was tooth; however, previous studies reported that the most common TFBs were fish and chicken bones in adults [3, 20], and magnets in children [3]. With the aging of the population, the number of people receiving dental treatment, and the frequency of TFBs, will increase. Dentists have recognized this problem and developed methods to prevent aspiration [21, 22]. Iatrogenic foreign-body aspiration accounted for 37% of all cases in this study. During intubation, teeth were aspirated in three cases, and pills, tracheostomy tubes, and cotton swabs were aspirated through the tracheostomy holes. As patients with risk factors for aspiration receive more medical interventions, the incidence of iatrogenic TFBs is expected to increase in the future. Therefore, more precautions are required to prevent aspiration during medical practices that increase aspiration risk.

Flexible bronchoscopy was successful in removing TFBs under light sedation in 91.3% of our patients. Several studies have reported the safety and effectiveness of TFB retrieval by flexible bronchoscopy [23, 24]. Delayed diagnosis of TFBs may increase the risk of lung resection [25]. TFBs may represent an especially significant problem for patients with mental or physical handicaps. Flexible bronchoscopy can be performed without general anesthesia [7]. In addition, removal by flexible or rigid bronchoscopy avoids the need of lung resection and preserves lung function. In our study, the most common cause of failure of removal the TFBs with flexible bronchoscopy was risk of bleeding due to inflammation with the surrounding tissues. In particular, rigid bronchoscopy could be a good option for these cases to remove TFBs and preserve lung volume. Also, flexible bronchoscopy is valuable in patients at high risk for general anesthesia [26].

Our study has several strengths and limitations. This data is representative, because foreign-body aspiration in adults is uncommon and we collected the data for past 20 years from all major university hospitals where bronchoscopy is available in our city. And the type of foreign body may vary depending on the table manners, medical environment, subjects’ age and times. This is the first data that iatrogenic foreign-body aspiration is the major cause of TFB. This is an important information that reminds the clinicians of the need to pay more attention in their practice. On the other hand, it has the limitations because of its retrospective observation study design; selective bias for only including patients who had taken bronchoscopy, a limitation of relying solely on the medical records left and not being able to investigate additional data.

Foreign-body aspiration can occur even in people without predisposing factors. The most common aspirated material in this study was tooth, and iatrogenic events accounted for 37% of TFBs. Flexible bronchoscopy with light sedation was relatively safe and effective for removing TFBs.

## References

[1] Baharloo F, Veyckemans F, Francis C, Biettlot M-P, Rodenstein DO. Tracheobronchial foreign bodies: presentation and management in children and adults. Chest. 1999;115(5):1357–62. doi: 10.1378/chest.115.5.1357 10334153

[2] Casalini AG, Majori M, Anghinolfi M, Burlone E, D’Ippolito R, Toschi M, et al. Foreign body aspiration in adults and in children: advantages and consequences of a dedicated protocol in our 30-year experience. J Bronchology Interv Pulmonol. 2013;20(4):313–21. doi: 10.1097/LBR.0000000000000024 24162114

[3] Mcguirt WF, Holmes KD, Feehs R, Browne JD. Tracheobronchial foreign bodies. The Laryngoscope. 1988;98(6):615–8. doi: 10.1288/00005537-198806000-00007 3374237

[4] Lan RS. Non-asphyxiating tracheobronchial foreign bodies in adults. Eur Respir J. 1994;7(3):510–4. doi: 10.1183/09031936.94.07030510 8013610

[5] Chen C-H, Lai C-L, Tsai T-T, Lee Y-C, Perng R-P. Foreign body aspiration into the lower airway in Chinese adults. Chest. 1997;112(1):129–33. doi: 10.1378/chest.112.1.129 9228368

[6] Rafanan AL, Mehta AC. Adult airway foreign body removal: what’s new? Clinics in chest medicine. 2001;22(2):319–30. doi: 10.1016/s0272-5231(05)70046-0 11444115

[7] Cunanan OS. The flexible fiberoptic bronchoscope in foreign body removal: experience in 300 cases. Chest. 1978;73(5):725–6. 639586

[8] Sehgal IS, Dhooria S, Ram B, Singh N, Aggarwal AN, Gupta D, et al. Foreign Body Inhalation in the Adult Population: Experience of 25,998 Bronchoscopies and Systematic Review of the Literature. Respir Care. 2015;60(10):1438–48. doi: 10.4187/respcare.03976 25969517

[9] Fields RT Jr, Schow SR. Aspiration and ingestion of foreign bodies in oral and maxillofacial surgery: a review of the literature and report of five cases. Journal of oral and maxillofacial surgery. 1998;56(9):1091–8. doi: 10.1016/s0278-2391(98)90263-4 9734773

[10] Hou R, Zhou H, Hu K, Ding Y, Yang X, Xu G, et al. Thorough documentation of the accidental aspiration and ingestion of foreign objects during dental procedure is necessary: review and analysis of 617 cases. Head & face medicine. 2016;12(1):1–8. doi: 10.1186/s13005-016-0120-2 27449659 PMC4957346

[11] Cameron SM, Whitlock WL, Tabor MS. Foreign body aspiration in dentistry: a review. J Am Dent Assoc. 1996;127(8):1224–9. doi: 10.14219/jada.archive.1996.0415 8803399

[12] Tiwana KK, Morton T, Tiwana PS. Aspiration and ingestion in dental practice: a 10-year institutional review. The Journal of the American Dental Association. 2004;135(9):1287–91. doi: 10.14219/jada.archive.2004.0404 15493393

[13] Ramos MB, Fernandez-Villar A, Rivo JE, Leiro V, Garcia-Fontan E, Botana MI, et al. Extraction of airway foreign bodies in adults: experience from 1987–2008. Interact Cardiovasc Thorac Surg. 2009;9(3):402–5. doi: 10.1510/icvts.2009.207332 19491125

[14] al-Majed SA, Ashour M, al-Mobeireek AF, al-Hajjaj MS, Alzeer AH, al-Kattan K. Overlooked inhaled foreign bodies: late sequelae and the likelihood of recovery. Respir Med. 1997;91(5):293–6. doi: 10.1016/s0954-6111(97)90033-0 9176648

[15] Yilmaz A, Akkaya E, Damadoglu E, Gungor S. Occult bronchial foreign body aspiration in adults: analysis of four cases. Respirology. 2004;9(4):561–3. doi: 10.1111/j.1440-1843.2004.00621.x 15612971

[16] Çaglayan S, Erkin S, Coteli I, Ontz H. Bronchial foreign body vs asthma. Chest. 1989;96(3):509–11. doi: 10.1378/chest.96.3.509 2766809

[17] Gordon L, Nowik P, Kesheh SM, Lidegran M, Diaz S. Diagnosis of foreign body aspiration with ultralow-dose CT using a tin filter: a comparison study. Emergency radiology. 2020;27(4):399–404. doi: 10.1007/s10140-020-01764-7 32152760 PMC7343722

[18] Hitter A, Hullo E, Durand C, Righini C-A. Diagnostic value of various investigations in children with suspected foreign body aspiration. European annals of otorhinolaryngology, head and neck diseases. 2011;128(5):248–52. doi: 10.1016/j.anorl.2010.12.011 22018977

[19] Kim M, Lee KY, Lee KW, Bae KT, MDCT evaluation of foreign bodies and liquid aspiration pneumonia in adults. Americal Journal of Roentgenology. 2008;190(4):907–15. doi: 10.2214/AJR.07.2766 18356436

[20] Limper AH, Prakash UB. Tracheobronchial foreign bodies in adults. Annals of internal medicine. 1990;112(8):604–9. doi: 10.7326/0003-4819-112-8-604 2327678

[21] Cossellu G, Farronato G, Carrassi A, Angiero F. Accidental aspiration of foreign bodies in dental practice: clinical management and prevention. Gerodontology. 2015;32(3):229–33. doi: 10.1111/ger.12068 24102914

[22] Cameron SM, Whitlock WL, Tabor MS. Foreign body aspiration in dentistry: a review. The Journal of the American Dental Association. 1996;127(8):1224–9. doi: 10.14219/jada.archive.1996.0415 8803399

[23] Oke V, Vadde R, Munigikar P, Bhattarai B, Agu C, Basunia R, et al. Use of flexible bronchoscopy in an adult for removal of an aspirated foreign body at a community hospital. Journal of community hospital internal medicine perspectives. 2015;5(5):28589. doi: 10.3402/jchimp.v5.28589 26486107 PMC4612481

[24] Mise K, Jurcev Savicevic A, Pavlov N, Jankovic S. Removal of tracheobronchial foreign bodies in adults using flexible bronchoscopy: experience 1995–2006. Surg Endosc. 2009;23(6):1360–4. doi: 10.1007/s00464-008-0181-9 18923871

[25] Duan L, Chen X, Wang H, Hu X, Jiang G. Surgical treatment of late-diagnosed bronchial foreign body aspiration: a report of 23 cases. The Clinical Respiratory Journal. 2014;8(3):269–73. doi: 10.1111/crj.12040 23848455

[26] Swanson KL, Prakash UB, Midthun DE, Edell ES, Utz JP, McDougall JC, et al. Flexible bronchoscopic management of airway foreign bodies in children. Chest. 2002;121(5):1695–700. doi: 10.1378/chest.121.5.1695 12006464

